# Agronomic biofortification increases grain zinc concentration of maize grown under contrasting soil types in Malawi

**DOI:** 10.1002/pld3.458

**Published:** 2022-11-03

**Authors:** Lester Botoman, Joseph G. Chimungu, Elizabeth H. Bailey, Moses W. Munthali, E. Louise Ander, Abdul‐Wahab Mossa, Scott D. Young, Martin R. Broadley, R. Murray Lark, Patson C. Nalivata

**Affiliations:** ^1^ Department of Crop and Soil Sciences Lilongwe University of Agriculture and Natural Resources Lilongwe Malawi; ^2^ Department of Agricultural Research Services Chitedze Agricultural Research Station Lilongwe Malawi; ^3^ School of Biosciences University of Nottingham Loughborough UK; ^4^ Inorganic Geochemistry, Centre for Environmental Geochemistry British Geological Survey Keyworth UK; ^5^ Rothamsted Research Harpenden UK

**Keywords:** agro‐fortification, Lixisols, maize, Vertisols, zinc deficiency, zinc‐enriched fertilizers

## Abstract

Zinc (Zn) deficiency remains a public health problem in Malawi, especially among poor and marginalized rural populations, linked with low dietary intake of Zn due to consumption of staple foods that are low in Zn content. The concentration of Zn in staple cereal grain can be increased through application of Zn‐enriched fertilizers, a process called agronomic biofortification or agro‐fortification. Field experiments were conducted at three Agricultural Research Station sites to assess the potential of agronomic biofortification to improve Zn concentration in maize grain in Malawi as described in registered report published previously. The hypotheses of the study were (i) that application of Zn‐enriched fertilizers would increase in the concentration of Zn in maize grain to benefit dietary requirements of Zn and (ii) that Zn concentration in maize grain and the effectiveness of agronomic biofortification would be different between soil types. At each site two different subsites were used, each corresponding to one of two agriculturally important soil types of Malawi, Lixisols and Vertisols. Within each subsite, three Zn fertilizer rates (1, 30, and 90 kg ha^−1^) were applied to experimental plots, using standard soil application methods, in a randomized complete block design. The experiment had 10 replicates at each of the three sites as informed by a power analysis from a pilot study, published in the registered report for this experiment, designed to detect a 10% increase in grain Zn concentration at 90 kg ha^−1^, relative to the concentration at 1 kg ha^−1^. At harvest, maize grain yield and Zn concentration in grain were measured, and Zn uptake by maize grain and Zn harvest index were calculated. At 30 kg ha^−1^, Zn fertilizer increased maize grain yields by 11% compared with nationally recommended application rate of 1 kg ha^−1^. Grain Zn concentration increased by 15% and uptake by 23% at the application rate of 30 kg ha^−1^ relative to the national recommendation rate. The effects of Zn fertilizer application rate on the response variables were not dependent on soil type. The current study demonstrates the importance of increasing the national recommendation rate of Zn fertilizer to improve maize yield and increase the Zn nutritional value of the staple crop.

## 1 INTRODUCTION

Micronutrient deficiencies, particularly zinc (Zn), are widespread in Malawi with large prevalence rate among women and children (Gupta et al., 2020; Siyame et al., 2013). Recent studies estimate that 62% of the Malawian population is Zn‐deficient (Likoswe et al., 2020; National Statistical Office [NSO], 2017), and this is likely to be larger in rural populations (Siyame et al., 2013; Tang et al., 2022). Zinc is an essential micronutrient which has important functions in all biological systems (Broadley et al., 2007). Its deficiency in humans is associated with multiple health problems that include immune system impairments, retarded physical growth and brain development among children under 5 years of age, and poor birth outcomes in women (Gibson, 2012; Krebs et al., 2014; Terrin et al., 2015). Various interventions, such as application of Zn‐enriched fertilizers, are possible means of reducing Zn deficiency in humans through increasing the concentration of Zn in the edible parts of crops (Joy, Stein, et al., 2015; Liu et al., 2017; Manzeke et al., 2014; White & Broadley, 2009). Field experiments were designed, based on pilot study data, to explore how to increase the Zn nutritional quality of maize grain in Malawi through agronomic biofortification; the protocol was published as a registered report (Botoman et al., 2020). The results are presented in this paper.

The main aim of this study was to assess the potential of agronomic biofortification by soil application of Zn‐enriched fertilizers to increase Zn concentration in the edible part of maize. Specifically, the study was conducted to: determine the extent to which the application of Zn‐enriched fertilizers to soils increases the concentration of Zn in grains; to examine differences in grain Zn concentration between soil types; and to determine how the effectiveness of agronomic biofortification differs between soil types. If the experiment provides evidence for an effect of agronomic biofortification on concentration of Zn in maize grain, then in the short term, this intervention could be a cost‐effective way to alleviate Zn deficiency among the rural population in Malawi.

## 2 MATERIALS AND METHODS

The protocol for the experiment was reported previously in detail (Botoman et al., 2020) and a brief description is provided here.

### 2.1 Materials

Maize was chosen as it is the principal staple cereal crop in Malawi. The maize variety used in the experiment was an F_1_ hybrid, SC 403, locally known as “Kanyani.” This variety was chosen because it is widely grown in Malawi, is early maturing, tolerates a wide range of environmental conditions and can mature in approximately 90 days. For Zn fertilizer treatments, a commercial grade ZnSO_4_·7H_2_O fertilizer (22% elemental Zn; M.R. Zinc, Dalview, South Africa) was used. Two soil types from the World Reference Base classification (IUSS Working Group WRB, 2006), Lixisols and Vertisols, were used for the study, representing agriculturally important soils of Malawi.

### 2.2 Description of the experimental sites

The study was conducted at Chitala, Chitedze, and Ngabu Agricultural Research Stations in Lilongwe, Salima, and Chikwawa districts, respectively, during the 2019–2020 cropping season. Prior to starting the experiment, soil samples were collected from five points randomly spaced across the whole experimental area of each soil type at each site, at a depth of 0–20 cm. The collected soil samples were thoroughly mixed, and a 500‐g composite sample was taken and analyzed for baseline soil characteristics (Table 1). Generally, the results show that the soils had a wide range of properties. Vertisols had larger mean values of pH, organic carbon, total nitrogen, and exchangeable bases across all sites, confirming that these soils are more fertile than Lixisols.

**TABLE 1:** Initial soil characteristics of the experimental sites

Soil property	Chitala		Chitedze		Ngabu	
	Lixisol	Vertisol	Lixisol	Vertisol	Lixisol	Vertisol
Organic carbon (%)	.98	1.46	1.33	1.64	.99	2.05
pH_(water)_	5.44	6.87	5.21	6.23	5.33	7.52
Total N (%)	.05	.16	.07	.23	.09	.39
Total Zn (mg kg^−1^)	88.5	89.0	77.5	97.0	155.0	196.5
DTPA Zn (mg kg^−1^)	1.60	3.34	.76	1.17	2.61	4.82
Available P (mg kg^−1^)	13.6	15.1	12.4	14.7	16.9	19.4
CEC (cmol_c_ kg^−1^)	18.1	26.8	11.5	24.5	17.5	29.5
Exchangeable Ca (cmol_c_ kg^−1^)	2.7	4.9	1.8	3.1	5.6	9.1
Exchangeable Mg (cmol_c_ kg^−1^)	.8	1.6	.6	1.5	1.3	1.9
Exchangeable K (cmol_c_ kg^−1^)	.5	.7	.1	.4	.9	1.2

Abbreviations: CEC, cation exchange capacity; DTPA, diethylenetriaminepentaacetic acid.

### 2.3 Zn fertilizer treatments, experimental design and statistical analysis

Different Zn application rates of 1, 30, and 90 kg ha^−1^ of elemental Zn were applied at the three‐leaf growth stage to all the plots at all sites. The gross plot size was five ridges, each 5 m long, with the net plot being the three middle ridges, each 3 m long. The ridges were spaced at 75 cm apart. All fertilizers were applied as a basal application by manually placing the fertilizers using the “spot” (or “dollop”) method at 10 cm depth and 12.5 cm away from the planting station at a right angle to the ridge axis, as typically practiced by farmers. The use of 1 kg Zn ha^−1^ was based on national recommended application rates for Zn fertilizers in Malawi (Ministry of Agriculture and Food Security [MoAFS], 2016). The use of 30 and 90 kg Zn ha^−1^ fertilizer rates in the experiment was informed by low Zn grain concentration in the pilot trial, where a lower maximum Zn application rate of 20 kg ha^−1^ was used (Botoman et al., 2020).

The three Zn fertilizer rates were applied to the three plots allocated within 10 complete randomized blocks at each of the six subsites (one on a Lixisol and one on a Vertisol at each of the three sites). The allocation of treatments to plots within blocks was done independently and at random using a script for the R platform (R Core Team, 2017). The choice of the number of blocks is critical to determine the sensitivity of the experiment to detect the true effect sizes of the fertilizer treatment, and because of this, we undertook a power analysis by simulation (Botoman et al., 2020).

Data analyses were conducted using the nlme package for the R platform (Pinheiro et al., 2021). A linear mixed model was used with a random effects structure to reflect how the fertilizer rate is randomized among plots within sets of blocks all within one subsite of a single soil type. A fixed effects model was used comprising main effects of fertilizer rate, soil type and their interaction. Further, the main effect of fertilizer rate was partitioned into linear and nonlinear components with an appropriate choice of orthogonal polynomials and similarly examined the partition of the soil‐fertilizer interaction into components based on these two components of the fertilizer effect. The output of the analysis tested the specific hypotheses concerning differences between soil types and fertilizer application rates with respect to response variable, with confidence intervals, of the effects of Zn fertilizer application on response variable at the 30 and 90 kg Zn ha^−1^ relative to the recommended rate of 1 kg Zn ha^−1^. After estimation of the model parameters histograms were plotted of the random effects estimates at each level, the marginal residuals were plotted against the fitted values (Figures S1–S4) and summary statistics (Tables S1–S4) were computed. These outputs were examined to evaluate the plausibility of the assumption of normally distributed errors with homogeneous variances. In the case of maize grain yields, grain Zn concentrations and uptake these assumptions were accepted. For Zn_HI_, these assumptions were not accepted and data were transformed using a natural log.

### 2.4 Trial implementation, data collection, and sample laboratory analysis

**FIGURE 1:**
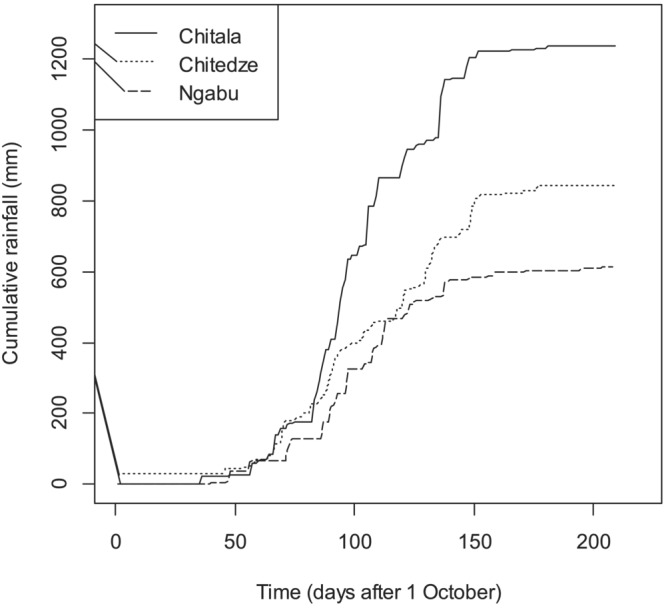
Rainfall distribution (mm) at Chitala, Chitedze, and Ngabu during the 2019–2020 cropping season

Maize was sown in December 2019 and harvested in April 2020 at Chitala and Ngabu and in May 2020 at Chitedze. At harvest, grain and stover samples were collected, prepared, and analyzed for grain and stover Zn concentrations as described by Botoman et al. (2020). A Certified Reference Material (CRM; Wheat flour SRM 1567b, National Institute of Standards and Technology, Gaithersburg, MD, US; 11.61 mg kg^−1^) and 12 operational blank digestions were used to determine the accuracy of the analyses and the limit of detection (LOD) for quality control. The Zn elemental recovery for SRM 1567b was 93%. To assess the benefit of Zn fertilizer application on maize yield, dry weight of maize grain (kg) was recorded from the net plots. Similarly, dry weight of stover (kg) was recorded and used to calculate Zn uptake by the crop and harvest index measures. Zinc uptake by the crop refers to the amount of Zn in the crop that is required to complete its life cycle. Zinc harvest index (Zn_HI_) is a ratio between Zn accumulated in the grain to that accumulated in the grain and stover combined (Fageria, 2014), expressed as a percentage. Daily rainfall data (mm) were also recorded using rain gauges stationed in each of the research stations where the experiment was conducted; results are presented in Figure 1. Generally, rainfall was well distributed at Chitala, fairly distributed at Chitedze and poorly distributed at Ngabu. Further, the difference in absolute quantities of rainfall is also apparent. No additional irrigation was used, in keeping with general agricultural practice in Malawi.

### 2.5 Measurements of the residual availability of zinc in soil

The residual benefit of soil applied Zn to subsequent crops for improved grain Zn nutritional quality has previously been noted (Boawn, 1974; Brennan & Bolland, 2007; Grewal & Graham, 1999; Mari et al., 2015). Given that large Zn application rates were used, a study on the residual benefit of Zn on subsequent maize crop was conducted. The maize crop was grown on the same plots and ridges without plowing or any added Zn. This information will be presented in our next report on the residual benefit of Zn for the maize crop grown in the subsequent cropping season.

## 3 RESULTS

### 3.1 Soil type and fertilizer effects on maize grain yields

The mean maize yields by treatment at all sites are shown in Figure 5a, along with their standard errors. No statistical inferences about the differences between soil types can be made on a site‐by‐site basis because soil type as a factor was not replicated at the sites. A linear mixed model (LMM) was fitted, as proposed by Botoman et al. (2020) with site, subsite within site, block, and a residual as random effects. The soil type and fertilizer treatment are considered as fixed factors because they were specifically chosen for the study. The main effect of Zn fertilizer rate was partitioned into linear and nonlinear components. At each site, a positive response of yield to Zn application was seen. The mean yield response to applying 30 kg ha^−1^ of Zn relative to the yield at 1 kg Zn ha^−1^ was about 660 kg ha^−1^ (11% higher over 1 kg ha^−1^). There was no further significant changes in yield when the Zn application rate was increased to 90 kg ha^−1^.

**FIGURE 2:**
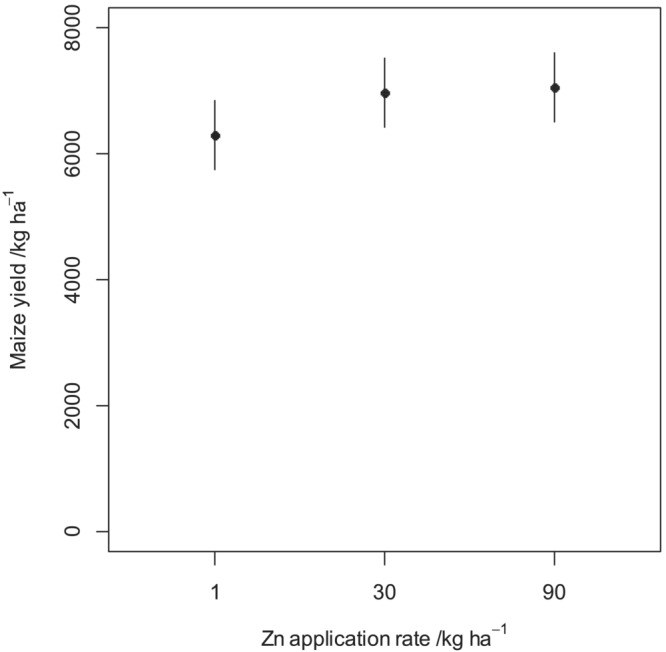
Mean maize grain yields obtained from the three experimental sites in response to Zn fertilizer application during the 2019–2020 cropping season. The error bars show the standard error of the mean (±SEM).

Table 2 shows the analysis of variance (ANOVA) for the yield response variable. Note that there is strong evidence for an effect of applied Zn, in the case of both a linear and nonlinear components. The linear component (*p* < .001) captures the positive effect of applied Zn on grain yield, while the nonlinear component (*p* = .005) corresponds to diminishing returns to the application of 90 kg ha^−1^ relative to the response at 30 kg ha^−1^ which can be seen in Figure 2. There was no evidence for differences among the soil types (*p* = .739) or for an interaction of Zn application rate with soil type. Thus, over all sites there was no difference in mean yield of maize grain between the two soil types, nor was there any evidence that the yield response to Zn differed between the soil types, either in the linear effect (*p* = .727) or the nonlinear effect (*p* = .278).

**TABLE 2:** ANOVA output table for maize grain yield, grain Zn concentrations, grain Zn uptake, and natural log of Zn harvest index at Chitala, Chitedze, and Ngabu

Factor	Num DF	Den DF	Grain yield		Grain Zn conc.		Grain Zn uptake		Zn_HI_	
			*F* value	*p* value	*F* value	*p* value	*F* value	*p* value	*F* value	*p* value
Soil type	1	2	.146	.739	.167	.723	.004	.956	.008	.936
Zn lin	1	116	15.941	<.001	39.16	<.001	51.993	<.001	64.075	<.001
Zn rem	1	116	8.11	.005	12.557	<.001	18.195	<.001	10.58	.002
Soil type ● Zn lin	1	116	.122	.727	.001	.982	.005	.944	1.089	.002
Soil type ● Zn rem	1	116	1.189	.278	.004	.953	.822	.366	3.05	.083

*Note*: A dot,●, denotes interaction; Zn lin = linear effect of Zn application rate and Zn rem = nonlinear effect of Zn application rate; Num DF = numerator degrees of freedom; Den DF = denominator degrees of freedom.

Table 3 shows the variance components from the LMM for each variable. For yield the between‐plot variance component was of comparable magnitude to the between‐site component, with rather smaller variances at intervening levels. This shows how crop yield is subject to multiple limiting factors which operate over a range of spatial scales.

**TABLE 3:** The variance component values from the LMM for each variable

Component and source	Maize grain yield (t^2^ ha^−2^)	Grain Zn conc. (observed^a^) (mg^2^ kg^−2^)	Grain Zn conc. (assumed^b^) (mg^2^ kg^−2^)	Grain Zn uptake (g^2^ ha^−2^)	Zn harvest index (ln%)
Between‐plot within block	.84	9.7	13.0	1120.0	.08
Between block	.07	2.0	.1	166.1	<.001
Between‐soil subsite within site	.09	4.9	2.0	<.001	.15
Between site	.81	7.2	3.0	195.7	<.001

^a^ Observed is the estimated variance component values of the present study.

^b^ Assumed is the estimated variance component values of the pilot study.

### 3.2 Effects of soil type and Zn fertilizer on maize grain Zn concentration and uptake

**FIGURE 3:**
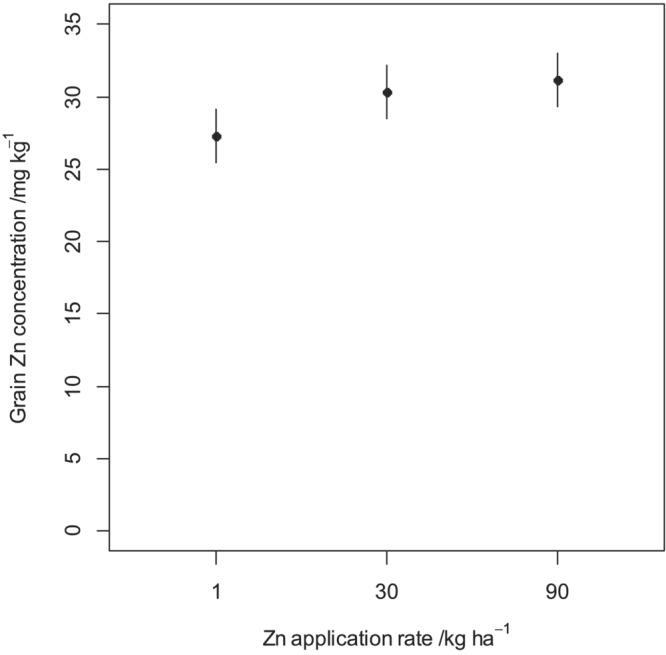
Mean grain Zn concentration at the three experimental sites in response to Zn fertilizer application during the 2019–2020 cropping season. The error bars show the standard error of the mean (±SEM).

**FIGURE 4:**
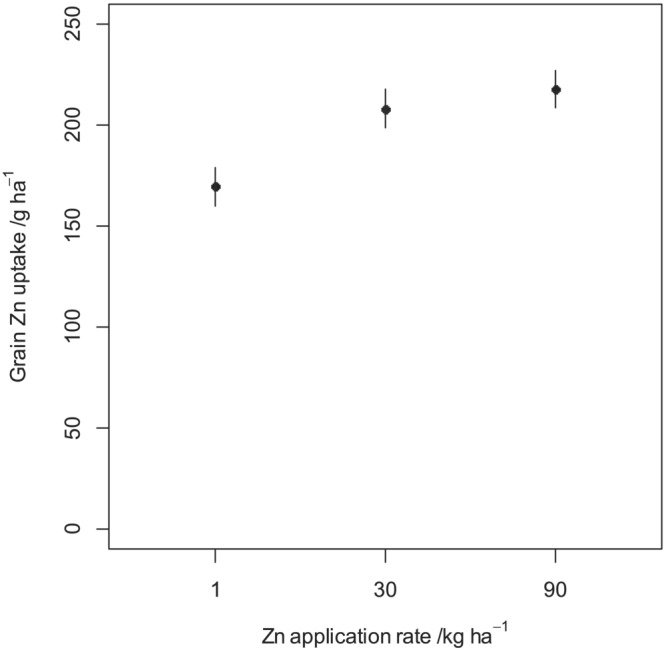
Mean grain Zn uptake at the three experimental sites in response to Zn fertilizer application during the 2019–2020 cropping season. The error bars show the standard error of the mean (±SEM).

**FIGURE 5:**
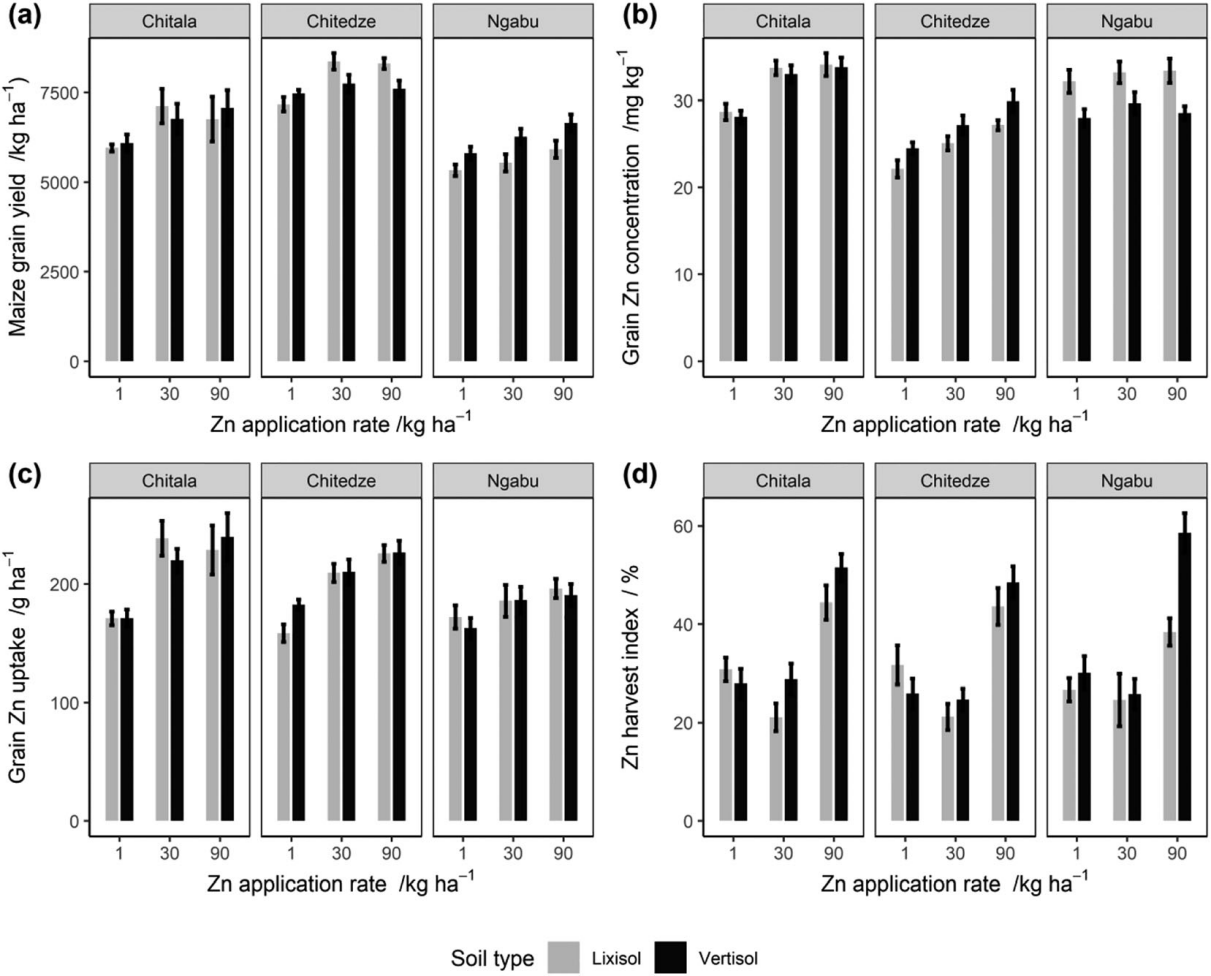
Effects of Zn fertilizer application and soil type on (a) maize grain yield, (b) grain Zn concentration, (c) grain Zn uptake, and (d) Zn harvest index at the three experimental sites during the 2019–2020 cropping season

The grain Zn concentrations and uptake for each fertilizer treatment at all experimental sites are presented in Figure 5b,c. This is accompanied by the standard errors calculated for each treatment level. Positive responses of grain Zn concentration and uptake to Zn application rate are apparent. Figures 3 and 4 show the mean grain Zn concentration and uptake, respectively, together with their standard errors for the three Zn fertilizer application rates as estimated in the LMM. Grain Zn concentration and uptake at 30 kg ha^−1^ were ~4 mg kg^−1^ (15% higher than 1 kg ha^−1^) and ~40 g ha^−1^ (23% higher than 1 kg ha^−1^), respectively, greater than at 1 kg ha^−1^; no further significant increases was observed when the Zn application was increased to 90 kg ha^−1^. In addition, there were noticeable differences in grain Zn concentration and uptake between the sites (Figure 5b,c).

The ANOVA for maize grain Zn concentration and Zn grain uptake are presented in Table 2. The results show that there was a significant response of maize grain Zn concentration and uptake to Zn fertilizer rate for the linear (*p* < .001) and nonlinear (*p* < .001) components of the response. Increasing the rate from 1 to 30 kg ha^−1^, resulted in a linear response while from 30 to 90 kg ha^−1^, yielded a nonlinear response. This means that increasing the Zn fertilizer rate from 1 to 30 kg ha^−1^ results in a proportional increase in maize grain Zn concentration and uptake, while from 30 to 90 kg ha^−1^ results in a proportionally smaller increases in grain Zn concentration and uptake. For both response variables, no significant differences (*p* = .723) in grain Zn concentration and (*p* = .956) in grain Zn uptake were observed among the soil types (Tables 2 and 3). In addition, there is no evidence that the linear and nonlinear response for grain Zn concentration depended on the soil type as the interaction of the soil type and linear effect (*p* = .982) and soil type and nonlinear effect (*p* = .953) are not significant. Similarly, there is no evidence that the linear and nonlinear response for grain Zn uptake depended on the soil type as the interaction of the soil type and linear effect (*p* = .944) and soil type and nonlinear effect (*p* = .366) were not significant. This suggests that maize grain Zn concentration and uptake over all sites is not different between soil types.

The variance component values from LMM for grain Zn concentration and uptake are presented in Table 3. For the grain Zn concentration, the between‐plot variance component value was larger than the between‐site variance component value. Similarly, the grain Zn uptake between‐plot variance component value was significantly higher than its between‐site variance component value. This shows how maize grain Zn concentration and uptake is affected by the spatial variation of Zn in soil at short distances within the same geographical location in Malawi, consequently contributing to the observed variations in maize grain Zn concentrations in Malawi (Botoman et al., 2022; Gashu et al., 2021). Table 3 also shows the variance components for each random effect which were assumed in the original power analysis (Botoman et al., 2020). Note that our estimates from the experimental data are of the same order of magnitude, suggesting that the approach of a power analysis based on estimates from pilot studies and survey data is a robust approach to the design of experiments of adequate power.

### 3.3 Soil type and Zn fertilizer effects on Zn harvest index

The mean Zn harvest indices (Zn_HI_), measures of Zn grain loading efficiency by each Zn treatment at all sites, are presented in Figure 5d, together with the standard errors estimated separately for each of these datasets. A LMM was used to analyze the effects of soil type, Zn treatment and their interaction on Zn_HI_, as described above. The outputs for testing normality of the residuals showed a skewed distribution and the response variable was transformed to natural logarithm prior to analysis. After the transformation, the residuals appeared consistent with the assumption of a normal distribution and homogeneity of variances (Figure S4).

**FIGURE 6:**
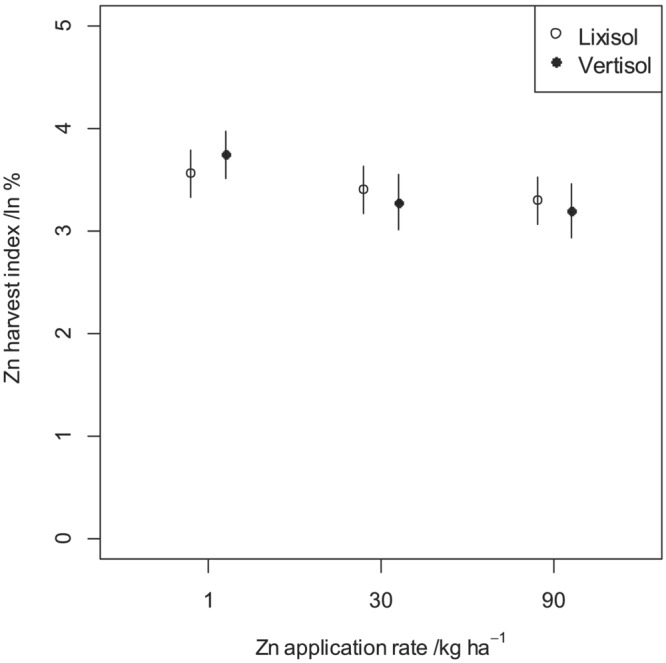
Mean Zn harvest index at the three experimental sites in response to Zn fertilizer application during the 2019–2020 cropping season. The error bars show the standard error of the mean (±SEM).

The mean Zn_HI_ decreased from 40% to 30% among the soil types in response to the increase of Zn application rate from 1 to 30 kg ha^−1^, and no effect was observed when the rate was further increased from 30 to 90 kg ha^−1^ (Figure 6). However, no statistical inference was made from the plot since soil type was not replicated within any experimental site. There are observed differences in Zn_HI_ between the sites. For example, there was about 8% more Zn loaded in the grain at Ngabu than Chitala and Chitedze. The variations in Zn_HI_ response between sites might be attributed to differences in soil physical and chemical charateristics (Table 1). Soils at Ngabu have a higher fertility status than those at Chitala and Chitedze.

The ANOVA for natural log of Zn harvest index is shown in Table 2. There was a significant effect of Zn_HI_ to Zn fertilizer rate for the linear (*p* < .001) and nonlinear (*p* = .002) components of the response. When the rate was increased from 1 to 90 kg ha^−1^, there was a negative effect for both linear and nonlinear responses. The linear component represents the proportional decrease in Zn_HI_ with increase in Zn application rate and the nonlinear component shows the minimal decrease in Zn_HI_ with the further increase in Zn application rate (Figure 6). The reduction in Zn_HI_ with application of Zn, with a large initial drop, and smaller effect from 30 to 90 kg ha^−1^ shows that Zn loading to the grain is negatively affected by the physiological response of the crop to Zn availability in the soil through reduction of Zn uptake by the crop roots. There is no evidence for the differences among the soil types (*p* = .936). However, the interaction between soil type and the linear component of the Zn rate is significant (*p* = .002). This suggests that the effect of Zn fertilizer on Zn_HI_ depends on soil type when the Zn application rate was increased from 1 to 30 kg ha^−1^.

The variance component values from the LMM for Zn_HI_ are presented in Table 3. The between‐plot variance component value is higher than the between‐site variance component value. This shows how the partitioning efficiency of Zn to the maize grain is influenced by the spatial variation of Zn in soil at distances of less than 100 km within the same geographical location and this affects the overall Zn concentration in the grain in the maize crop.

## 4 DISCUSSION

### 4.1 Zinc fertilizer, but not soil type, influenced maize grain yields

Zinc plays important physiological roles in maize and its deficiency can reduce grain yields by up to 10% (Joy, Stein, et al., 2015). Several studies report a positive response of maize grain yields to the application of Zn fertilizer (Manzeke et al., 2014; Palai et al., 2020; Stewart et al., 2021). For example, Liu et al. (2020) reported maize yield increases of about 4% to 17% with soil application of Zn fertilizer in China. Similarly, the findings of this study show that application of Zn fertilizer resulted in significant increases in maize grain yields. Soil application of Zn fertilizer of 30 kg ha^−1^ increased maize grain yields by 11% over the national recommended Zn fertilizer rate of 1 kg ha^−1^. The additional grain produced when Zn fertilizer rate was increased from 1 to 30 kg ha^−1^ was ~660 kg ha^−1^. This translates to a minimum annual benefit (minimum additional income for the farmer) of about MK30,000 ha^−1^ (~$40 ha^−1^) based on the cost of Zn fertilizer and return on yield. The cost of Zn fertilizer was calculated based on commercial price (MK960 kg^−1^) and estimated quantities of 135 and 4.5 kg for 30 and 1 kg ha^−1^, respectively. The return on yield was calculated using the minimum government maize price of MK220 kg^−1^ and obtained maize yields at respective Zn fertilizer application rates. This shows that some benefit is realized in the first year. However, the annual benefit might be higher than the estimated as the price of maize varies with location. Further, the benefit of Zn fertilizer application could increase from the second year due to residual benefit of Zn in soil (Boawn, 1974; Brennan & Bolland, 2007).

In Malawi, Zn fertilizer application to improve maize crop yields is recommended (MoAFS, 2016). The ability to add the Zn to the fertilizer blend already recommended by the Government of Malawi further means that this approach does not create additional labor or other time costs for farmers, compared with current practice. This may also incur a smaller price differential than purchase of Zn fertilizer. Some researchers have suggested that the increase in grain yield with the application of Zn fertilizer is due to increase in kernel density (Abunyewa & Mercie‐Quarshie, 2004; Liu et al., 2020; Potarzycki, 2010). The findings of the present study further demonstrate that the positive response of grain yield to Zn fertilizer is not dependent on soil type. This suggests that a Zn fertilization program to increase maize grain yields in Malawi can be implemented on these tested soil types with a uniform appropriate Zn fertilizer rate. However, future studies should consider investigating other soil types and agro‐ecological zones.

### 4.2 Zinc fertilization, but not soil type, influenced maize grain Zn concentration and uptake

The application of Zn fertilizer increased maize grain Zn concentration and uptake. The application of 30 kg ha^−1^ Zn compared with the current national recommendation rate of 1 kg Zn ha^−1^ increased grain Zn concentration by 15%. Manzeke et al. (2014) and Manzeke‐Kangara et al. (2021) reported larger increases of maize grain Zn of up to 67% with combined application of Zn fertilizer (11 kg Zn ha^−1^), mineral fertilizers (90 kg N ha^−1^ and 26 kg P ha^−1^), and locally available organic resources (5 t ha^−1^) were observed. The inconsistency in results of the current study with those reported by Manzeke et al. (2014) may be due to effect of different growing conditions. Maize in this study is high yielding as it is conducted at a research station site rather than on more infertile smallholder soils and this might explain the observed differences in grain Zn concentration. The overall maize grain Zn uptake in the present study ranged between 170 and 218 g ha^−1^. Manzeke et al. (2014) reported maize grain Zn uptake of between 7.7 and 115 g ha^−1^ when Zn fertilizer was applied in combination with organic nutrient resources and mineral fertilizers. On average, ~4 mg kg^−1^ increase in grain Zn concentration was observed when Zn fertilizer rate was increased from the national recommendation rate of 1 kg ha^−1^. This increase might benefit the Zn dietary intake of people in rural areas who rely heavily on maize as the major staple crop (Joy, Kumssa, et al., 2015; Joy, Stein, et al., 2015; Manary et al., 2002). The reported data further showed that the positive response of Zn fertilizer on grain Zn concentration and uptake did not depend on soil type. Based on the findings of the present study, an agronomic biofortification program of maize in Malawi might be implemented on these two soil types using a blanket Zn fertilizer application, without adjustment of the fertilizer application common practice used by farmers in the country, and leading to a more nutritious, harmonized grain Zn concentration across these areas of Malawi.

### 4.3 Zinc fertilization and interaction effect with soil type influenced maize grain Zn harvest index

Zinc harvest index (Zn_HI_) measures the efficiency of the crop in loading Zn into edible parts. The current study shows that soil type had no effect on Zn_HI_ while varying the application rate of Zn fertilizer resulted in significant decreases in Zn_HI_. For example, 1, 30, and 90 kg ha^−1^ yielded mean grain Zn loading efficiencies of 40%, 30%, and 30%, respectively. Zinc loading to the grain decreased by 10% when the rate was increased to 30 and 90 kg ha^−1^. Furthermore, the study shows that the interaction of soil type and the linear component of the response influenced Zn_HI_. This means that the combined effect of soil type and Zn fertilizer application influenced the loading efficiency of Zn to the maize grain, while both grain Zn concentration and grain yield increased. The present findings are consistent with those reported by Liu et al. (2019) where Zn_HI_ of maize grown in China under field conditions decreased from 74% to 52% when Zn fertilizer rates were increased from 2.3 to 34.1 kg ha^−1^. Similarly, Erenoglu et al. (2011), working on wheat, reported that the percentage of Zn translocated from the root to the shoot decreased as the Zn application rate increased. A possible explanation for this might be that the delivery of Zn to the root xylem is kinetically challenged, for example, xylem loading and unloading of Zn could be suppressed by high levels of available Zn (Curie et al., 2009; Palmer & Guerinot, 2009).

## 5 CONCLUSION

The current study provides evidence of the effectiveness and efficiency of agronomic biofortification through application of mineral Zn fertilizers to a range of soils in Malawi. The results showed that Zn fertilizer application increases the Zn nutritional value of maize grain which can help to meet dietary Zn requirements in humans. In the short term, this strategy could be a cost‐effective way to alleviate Zn deficiency among the rural populations of developing countries such as Malawi. Further data is needed on residual effects and to fully optimize the application rate based on these data. Increasing Zn concentration in staple food crops reduces rural households' nutritional vulnerability emerging from climatic and economic shocks, without requiring dietary change. The results of the study provide a basis for taking evidence‐based policy direction in the agricultural sector in Malawi and other countries in addressing Zn deficiency.

## CONFLICT OF INTEREST

The authors declare no competing interests.

## AUTHOR CONTRIBUTIONS

LB conceptualized the study as part of his PhD studies, under the supervisory guidance of PCN (lead supervisor, LUANAR), JGC, MWM, EHB, ELA, RML, AWM, SDY, and MRB. LB conducted experimental work. RML led on statistical guidance. LB, PCN, RML, and MRB wrote the primary draft of the paper with editing and reviewing inputs from all other authors.

## Supporting information


**Data S1.** Supporting Information

**Table S1.** Summary statistics of data on maize grain yield (kg ha^−1^)
**Figure S1.** Residuals against fitted values and histogram for the residuals of the random effects for maize grain yield
**Table S2.** Summary statistics of data on maize grain Zn concentration (mg kg^−1^)
**Figure S2.** Residuals against fitted values and histogram for the residuals of the random effects for concentration of Zn in grain
**Table S3.** Summary statistics of data on maize grain Zn uptake (g ha^−1^)
**Figure S3.** Residuals against fitted values and histogram for the residuals of the random effects for grain Zn uptake
**Table S4.** Summary statistics of data on Zn harvest index (ln %)
**Figure S4.** Residuals against fitted values and histogram for the residuals of the random effects for Zn harvest index

## Data Availability

The data generated for this study are available in the Supporting Information.
