# Supplementary material for: Agronomic biofortification increases grain zinc concentration of maize grown under contrasting soil types in Malawi
Source: Plant Direct. 2022 Nov 3;6(11):e458. doi: 10.1002/pld3.458 (PMC9631327; doi:10.1002/pld3.458)
Supplement: Supplementary file 2 — Table S1. Summary statistics of data on maize grain yield (kg ha−1) Figure S1. Residuals against fitted values and histogram for the residuals of the random effects for maize grain yield Table S2. Summary statistics of data on maize grain Zn concentration (mg kg−1) Figure S2. Residuals against fitted values and histogram for the residuals of the random effects for concentration of Zn in grain Table S3. Summary statistics of data on maize grain Zn uptake (g ha−1) Figure S3. Residuals against fitted values and histogram for the residuals of the random effects for grain Zn uptake Table S4. Summary statistics of data on Zn harvest index (ln %) Figure S4. Residuals against fitted values and histogram for the residuals of the random effects for Zn harvest index [file PLD3-6-e458-s002.docx]

**Supplementary information**

**Exploratory analysis of model residuals**

**Table S1**. Summary statistics of data on maize grain yield (kg ha^−1^)

Mean Median Quartile.1 Quartile.3 Variance SD Skewness

Experiment-level 0 -80.67 -811.85 981.73 1483011.3 1217.79 -0.30

Site-level 0 37.70 -495.08 621.50 898080.9 947.67 -0.50

Subsite-level 0 28.01 -474.83 537.83 856257.6 925.34 -0.51

Block-level 0 26.86 -421.72 516.88 747018.2 864.30 -0.51

Octile skewness Kurtosis No. outliers

Experiment-level 0.10 -0.64 0

Site-level 0.00 0.52 0

Subsite-level 0.01 0.71 0

Block-level -0.02 0.73 0


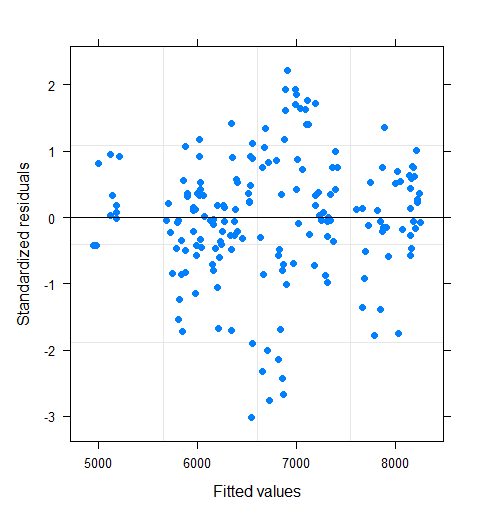


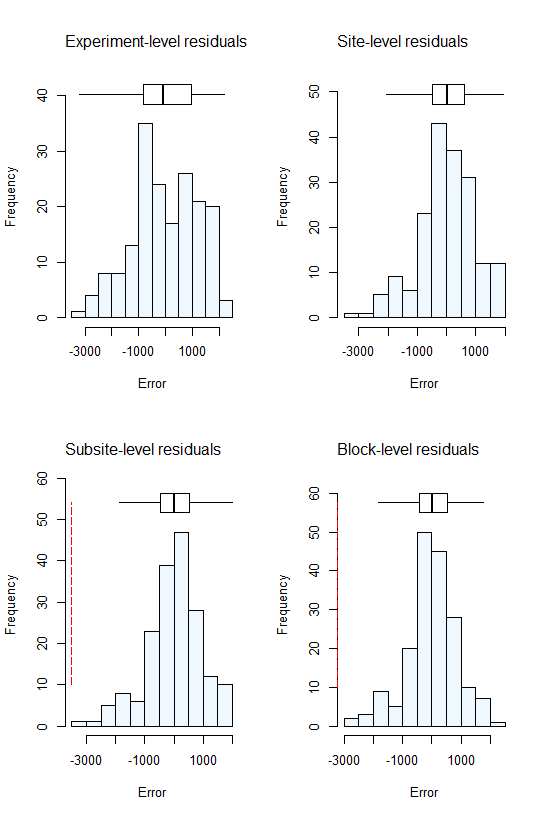


**Figure S1.** Residuals against fitted values and histogram for the residuals of the random effects for maize grain yield

**Table S2**. Summary statistics of data on maize grain Zn concentration (mg kg^−1^)

Mean Median Quartile.1 Quartile.3 Variance SD Skewness

Experiment-level 0 0.40 -3.23 2.95 19.49 4.41 0.13

Site-level 0 0.12 -2.52 2.03 13.35 3.65 0.36

Subsite-level 0 -0.11 -2.42 1.89 11.05 3.32 0.25

Block-level 0 -0.07 -1.89 2.03 8.10 2.85 0.14

Octile skewness Kurtosis No. outliers

Experiment-level -0.07 -0.32 0

Site-level -0.04 0.19 0

Subsite-level 0.01 -0.07 0

Block-level 0.06 -0.03 0


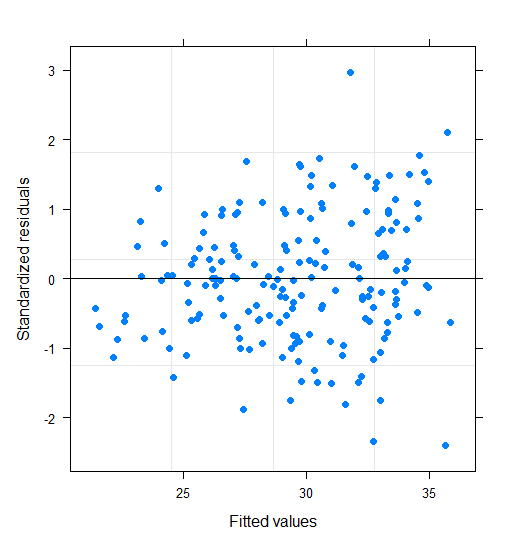


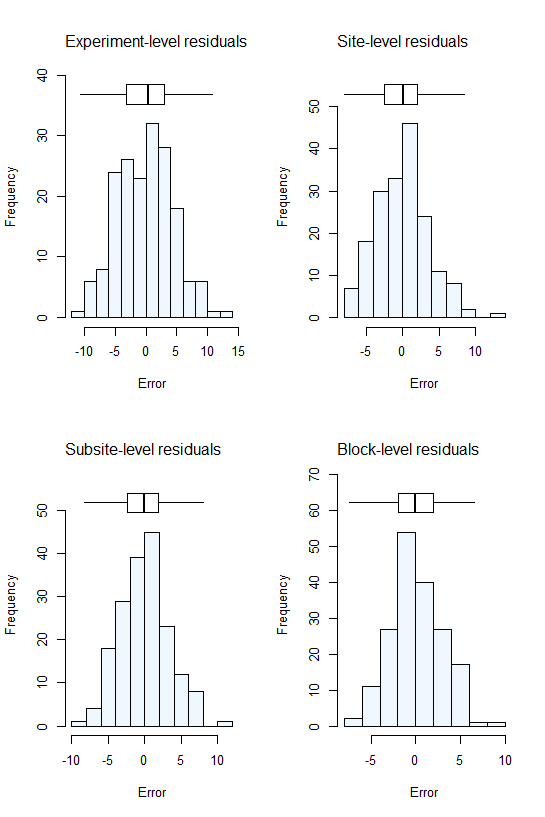


**Figure S2**. Residuals against fitted values and histogram for the residuals of the random effects for concentration of Zn in grain

**Table S3**. Summary statistics of data on maize grain Zn uptake (g ha^−1^)

Mean Median Quartile.1 Quartile.3 Variance SD Skewness

Experiment-level 0 -2.13 -24.51 21.03 1381.61 37.17 0.30

Site-level 0 -1.26 -21.73 20.22 1234.50 35.14 0.18

Subsite-level 0 -1.26 -21.73 20.22 1234.50 35.14 0.18

Block-level 0 -1.72 -17.91 18.02 969.59 31.14 0.16

Octile skewness Kurtosis No. outliers

Experiment-level 0.07 0.59 0

Site-level 0.03 0.57 0

Subsite-level 0.03 0.57 0

Block-level 0.06 0.65 0


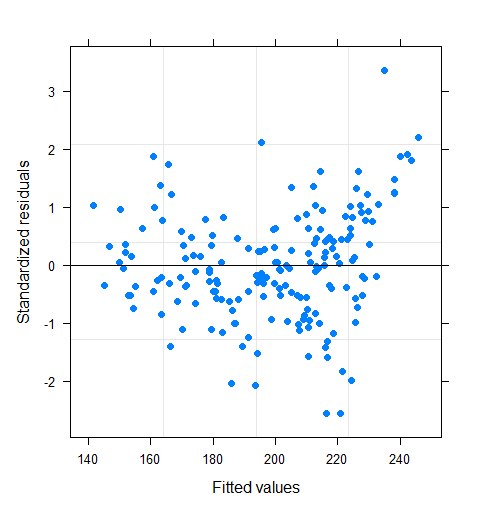


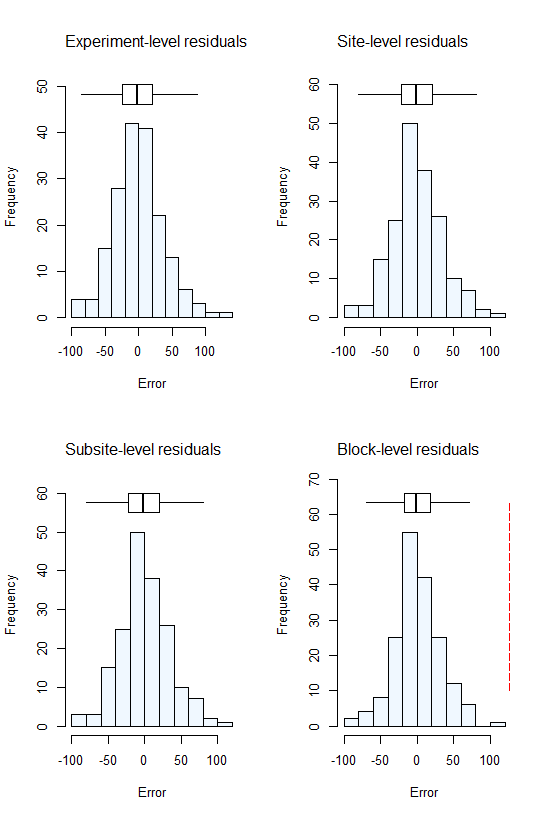


**Figure S3**. Residuals against fitted values and histogram for the residuals of the random effects for grain Zn uptake

**Table S4**. Summary statistics of data on Zn harvest index (ln %)

Mean Median Quartile.1 Quartile.3 Variance SD Skewness

Experiment-level 0 -0.03 -0.34 0.32 0.22 0.47 0.08

Site-level 0 -0.02 -0.28 0.28 0.17 0.41 0.11

Subsite-level 0 -0.01 -0.16 0.18 0.08 0.28 -0.19

Block-level 0 -0.01 -0.16 0.18 0.08 0.28 -0.19

Octile skewness Kurtosis No. outliers

Experiment-level 0.13 -0.56 0

Site-level 0.10 -0.46 0

Subsite-level 0.10 0.36 0

Block-level 0.10 0.36 0


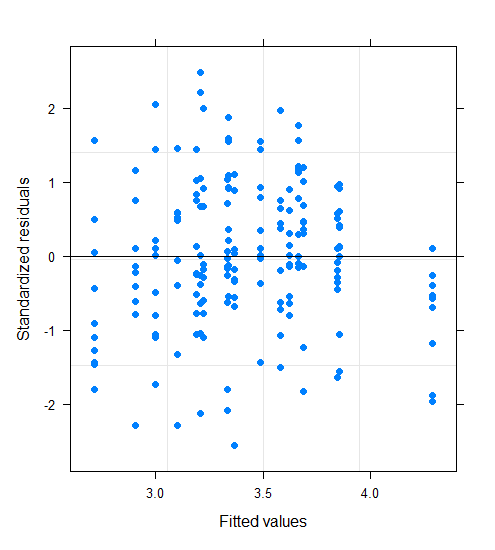


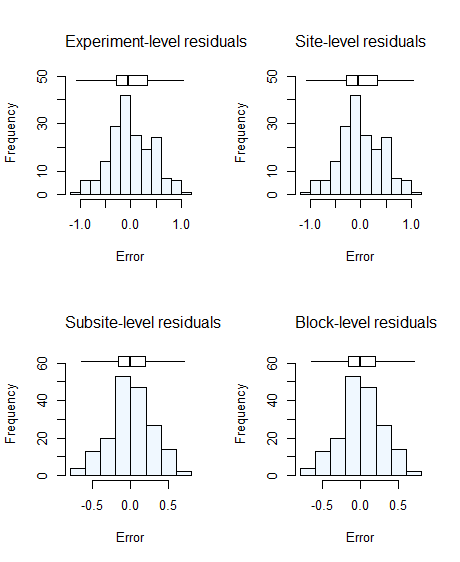


**Figure S4**. Residuals against fitted values and histogram for the residuals of the random effects for Zn harvest index
